# Microbial succession of lignocellulose degrading bacteria during composting of corn stalk

**DOI:** 10.1080/21655979.2021.2002622

**Published:** 2021-12-11

**Authors:** Fengmei Shi, Hongjiu Yu, Nan Zhang, Su Wang, Pengfei Li, Qiuyue Yu, Jie Liu, Zhanjiang Pei

**Affiliations:** a Heilongjiang Academy of Black Soil Conservation and Utilization, Harbin, China; b Key Laboratory of Combining Farming and Animal Husbandry Ministry of Agriculture, P. R. China, Harbin, China; c Key Laboratory of Energy Utilization of Main Crop Stalk Resources, Harbin, China

**Keywords:** Biodiversity, compost, corn stalk, bacteria, lignocellulose

## Abstract

The discarding and burning of corn stalks in the fields after harvesting lead to environmental pollution and waste of resources. Composting is an effective way to disposal of the crop straws. Composting is a complex biochemical process and needs a detailed study in cold region. Hence, the succession process of bacteria and *Actinomycetes* in the process of corn stalk composting in cold region was studied by 16SrRNA. *Alpha* diversity analysis showed that the detection results could represent the real situation. The bacterial community diversity from high to low was F50 > F90 > F0 > F10 > F20. The results of beta analysis showed that F20 and F50 had the most similar microbial structure at the phylum level, and the difference between F0 and F20 was the largest. The dominant microbes changed from *Proteobacteria* and *Bacteroidetes* in F0 in heating stage to *Firmicutes* and *Proteobacteria, Actinobacteria* and *Firmicutes* in F10 during early high temperature stage, and *Actinobacteria, Proteobacteria* and *Bacteroidetes* in cooling and post composting phases. *Actinobacteria* and *Firmicutes* were the dominant bacteria in the whole composting process. In the composting process, the microbial community was mainly involved in amino acid metabolism related to nitrogen transformation and carbohydrate metabolism related to lignocellulose degradation. Lignin and hemicellulose were mainly degraded in thermophilic stage. The conversion of nitrogen and degradation of cellulose occurred mainly in the early stages of composting. The research will be helpful to understand the biochemical process of composting in cold region.

## 1. Introduction

Corn is widely cultivated and about 192 million hectares of corn was planted in 2019 all over the world. China is the second largest corn producer and more than 2 billion tons of corn stalk was produced which need proper usage [1,2]. Composting is regarded as one of the effective ways to utilize stalk in China or even in the other countries [3,4]. Composting is a complex biochemical process. Fungi, bacteria and *Actinomycetes* participate in the degradation of the organic substances and humus are finally formed [5–7]. The species and relative abundance of microorganism affecting the speed and quality of composting, are impacted by the properties of substrates, the composting stages and the operation parameters of composting [8–10]. Thermophilic microorganisms such as *Actinobacteria* and *Proteobacteria* play key roles at high temperature, while mesophilic microorganisms become dominant at heating, cooling and maturity stages [6,11]. The dominant microorganisms are diverse when different composting substrates are used [12,13]. If the lignocellulose content in composting substrate is high, *bacteria* and *actinomycetes* will be the dominant participants in the composting process [14,15].

Industrial composting has high efficiency and good quality of the end product [16,17]. It is considered that a better living conditions can be supplied for microorganisms during industrial composting [18]. However, its application and popularization are restricted by the high cost of corn stalk removal, storage after leaving the field and the investment of certain equipment [17]. Traditional composting is operated outdoors with the advantages of economy and simple operation, but it is more sensitive to the climate [19]. It starts up difficultly or fails in winter due to massive heat loss in cold or frigid region such as in Heilongjiang Province [20]. The problem can be resolved by adding microbial agents consisting of several bacteria with different functions on organic materials during composting. Meanwhile, it is necessary to have a comprehensive understanding of the succession of microbial community and their function during composting of corn stalk in cold region. There were more studies focusing on microbial community succession and a few researches on microbial functions during composting [8–10,14,18]. Ding et al [18] studied the succession of microbial community during the composting of agricultural waste (the mixture of lawn grass, tomato stalk and cattle dung) and predicted their function using PICRUSt (phylogenetic investigation of communities by reconstruction of unobserved). The amino acid metabolism and carbon metabolism were the highest relative abundance in bioreactor composting and traditional composting in the heating phase, respectively. The carbohydrate metabolism and amino acid metabolism were two largest metabolism groups. They found that environmental conditions affected the microbial community function [18]. So the microbial community function and succession may be different because of different climate and substrates [18]. So far, metabolism pathway and interactions during traditional composting of corn stalk in clod region are still lack of research. Therefore, 16S rRNA (16S ribosomal ribonucleic acid) method was used to study the microbial succession, and the microbial metabolic metabolism pathway were predicted by PICRUSt. It is possible to screen out new strains with various degradation functions combined PCR molecular biological techniques according to the results of this study. Then efficient microbial agent can be developed to accelerate composting and improve the quality of compost.

## 2. Materials and methods

### 2.1. Stalk composting

The field composting experiments were carried out in Lanxi County, Heilongjiang Province in early April 2019 and lasted for 90d (days). The corn stalks were composted as follows [19]: about 2 tons of corn stalk (moisture content is about 11%) were collected, smashed into pieces with the length of 2 cm. A block with the size of 2.0 m × 1.5 m × 1.8 m was piled up including 7 layers with the stalk pieces, and covered with membranes. The microbial agent and urea (N wt%≥46.2%) were scattered on each stalk layer. The proportion of corn stalk, microbial agent and urea is 90.83%, 0.09% and 9.08%. The water content of the composting piles were adjusted to 55%. Three compost piles were built and turned over after 30d. The temperatures were measured and recorded everyday. The samples were collected every 10d, sealed in a plastic bag and stored in the refrigerator at −80°C. Some of the samples were air-dried for analysis.

### 2.2. Test method

The temperature of the compost pile was measured using a telethermometer (TPJ21-G, Hangzhou) at 22 am. The compost samples were extracted with deionized water for 1 h according to water/fertilizer of 10:1, then the EC and pH were measured by a pH meter (PHS-3 C, Shanghai) and a conductivity meter (DDB-303A, Shanghai) [19]. The absorbance of the extract solution was measured at 465 nm and 654 nm using an ultraviolet spectrophotometer (SP-752, Shanghai), which was denoted as E_4_ and E_6_, respectively [19]. Then E_4_/E_6_ was calculated according to the ratio of E_4_ to E_6_ [19]. The moisture content and VS were determined by drying method and muffle furnace method, respectively [19].

### 2.3. Study on bacterial succession and metabolic pathway

16S rRNA high throughput sequencing technique was used to study the succession of bacterial community in the stalk composting samples of 0d, 10d, 20d, 50d and 90d named as F0, F10, F20, F50 and F90 according to the temperature changes of the composting piles [19]. Clean data was obtained by PCR (polymerase chain reaction) amplification, library construction, fragment size and concentration detection, sequencing and sequencing of qualified gnomic DNA (deoxyribonucleic acid) samples [18]. Then OTUs (operational taxonomic units) were obtained by clustering the clean data [18]. Finally, the alpha diversity indexes and rarefaction curves were analyzed based on the results of OTUs via sofware mother(v.1.31.2) and R(v3.1.1), respectively [21,22]. The sequence number of each sample was unified. A new OTU table biomfile was generated by randomly selecting the sequence number of the sample with the least sequence number from all the samples and the file was used to calculate the distance of beta diversity via software QIIME (v1.80) [23]. Then the microbial metabolic metabolism pathway was predicted by PICRUSt according to the 16S rRNA result. Firstly, the influence of copy number of 16S marker gene in species genome was removed. Then the KO (KEGG Orthology) corresponding to the OTU was fixed from the Greengene ID, and the abundance of KO was calculated. In addition, PICRUSt was used to obtain the information of Metabolic Pathway at 3 levels [18,24].

Excel 2007 andOrigin2015 were used to handle the data and draw the figures, respectively.

## 3. Results and discussion

The compost properties and succession of the composting samples were analyzed. The results will be helpful to further understand the composting process of corn stalks in cold region.

### 3.1. Changes in physical and chemical properties

**Figure 1.**
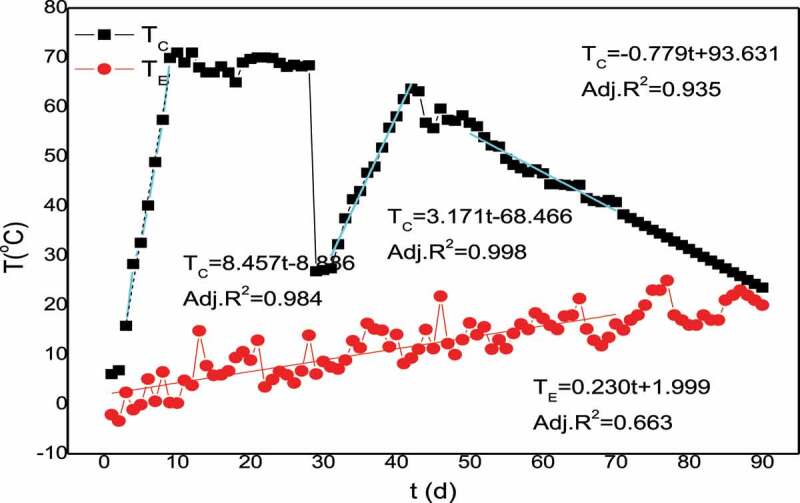
Temperature changes during corn stalk composting process [19]

The Physical and chemical properties of the corn stalk composting samples were shown in Table 1. The temperature inside the pile on the first day was 6.1°C, reached 71°C on the 10th day and the temperature was maintained until the 30th day. The temperature dropped to 27.1°C after the pile was turned over and rapidly rose to 58.1°Con the 40th day. On the 70th day, the temperature in the reactor body was below 40°C, and on the 90th day, the temperature reached 29.9°C, indicating that the mature of the corn stalk was complete.The water content was in the range of 40%-70% in the composting process, and the pH value was maintained in the range of 7.0–9.3. On the 90th day, the VS of corn stalk compost reached 32.36% lower than 56.78% of cattle manure compost in other literature [15]. It may be explained that the composting temperature reached 70°Cand kept for a long time, which led to excessive degradation of organic matter. The EC of the sample at 40d was less than 2.5 ms /cm, indicating that the compost was safe and could be applied safely [6]. Figure 1

**Table 1.** Physical and chemical properties of corn stalk during compost

Period	Water content (%)	pH	E_4_/E_6_	EC (μS/cm)	VS (Basis on dry matter) (%)
d0	53.06	7.3	1.34	1372	91.08
d10	60.48	9.29	2.34	403	81.83
d20	58.60	8.95	1.73	2890	73.23
d30	53.45	8.06	1.34	3130	67.93
d40	66.50	8.85	1.26	744	65.81
d50	51.31	8.72	1.93	684	53.47
d60	50.72	8.80	0.88	619	47.32
d70	47.60	8.92	1.64	551	42.27
d80	46.15	7.83	1.49	507	37.19
d90	44.40	7.05	1.35	448	32.36

### 3.2. Microbial succession and metabolic pathway

#### 3.2.1. Alpha diversity analysis

**Figure 2.**
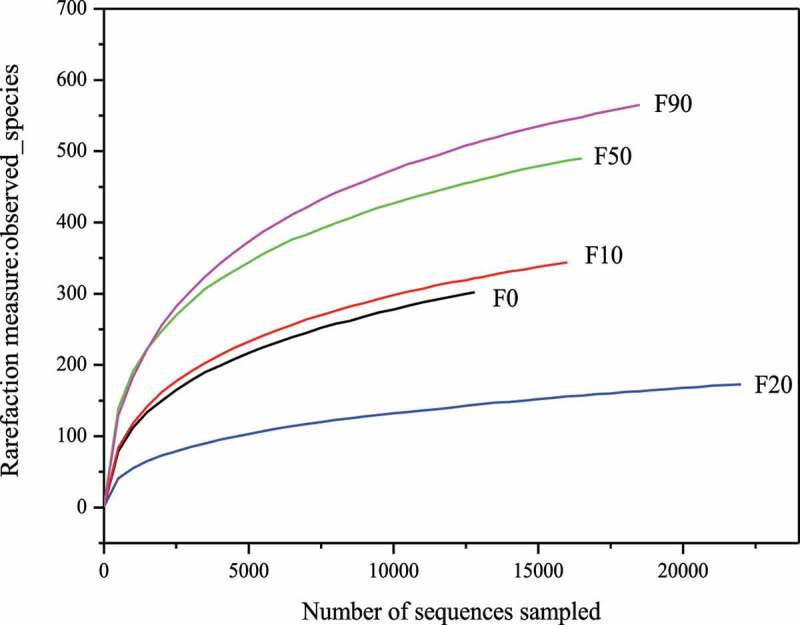
Rarefaction curve of the f corn stalk composting samples

Alpha diversity can quantitatively reflect the number of bacteria spices and their relative abundances in the samples. The Observed species index, Shannon index, Simpson index and Chao1 index were often used to describe alpha diversity [6,15,18]. The rarefaction curves and measure indexes of the stalk composting samples were showed in Figure 2 and Table 2, respectively. It can be seen that the observed-species index curves became flat when number of sequences sampled was larger than 5000 and each sample coverage was larger than 0.99, which meant that almost all of the bacteria in the samples were detected and the analysis results were reliable. Chao1 indexes were between 233.239–677.84, and the samples with the relative abundance from high to low was F90, F50, F10, F0 and F20. The diversity of bacterial community was F50 > F90 > F0 > F10 > F20 according to the Shannon and Simpson indexes.

**Table 2.** Rarefaction measure indexes of corn stalk composting samples

Sample Name	Chao1	Shannon	Simpson	Coverage
F0	429.66	3.27	0.10	0.992
F10	443.19	3.04	0.18	0.994
F20	233.239	2.27	0.21	0.998
F50	596.68	4.57	0.02	0.993
F90	677.84	4.30	0.04	0.992

#### 3.2.2. Beta diversity analysis

**Figure 3.**
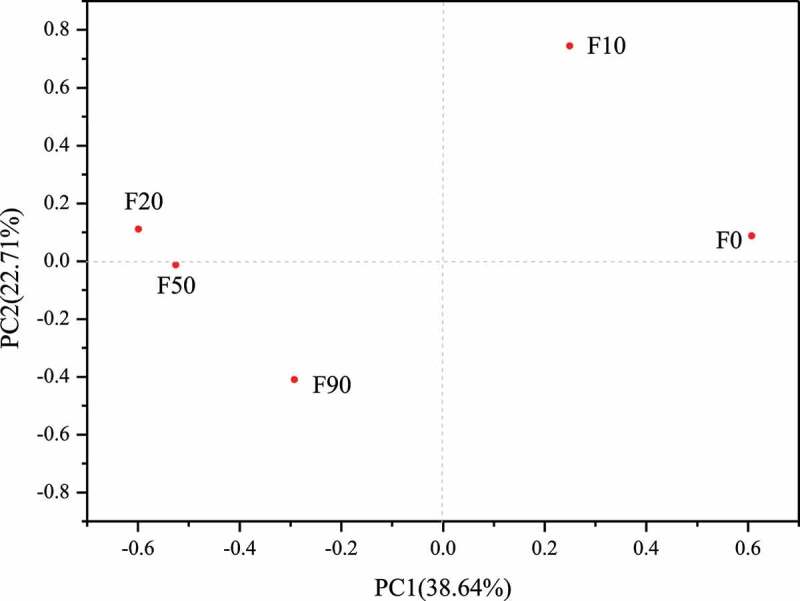
Principal components factoring analysis of corn stalk composting samples

Different from alpha diversity analysis, beta diversity analysis is mainly used to compare the composition and relative abundance of microbe species in different samples. That is, alpha diversity analysis describes the microorganisms in the same sample, while beta diversity analysis describes the microorganisms in different samples. Principal components analysis (PCA) is used to reflect the microbial community differences between different samples. The microbial community structure of the two samples is similar when the distance between the samples is short. As showed in Figure 3, the five samples were laid in three quadrants, in which F0 and F10, F20, F50 and F90 laid in the first, second and third quadrant, respectively. But F20 was closest to F50 and farthest from F0 among the five samples. The conclusion can be obtained that the microbial community structure in F20 was the most similar to that in F50 were and most different from that in F90.

**Figure 4.**
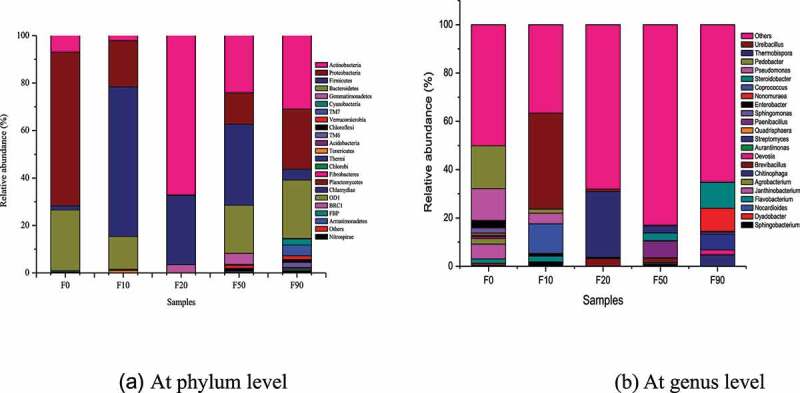
Bacterial community in compositing samples at phylum (a) and genus (b) level

**Figure 5.**
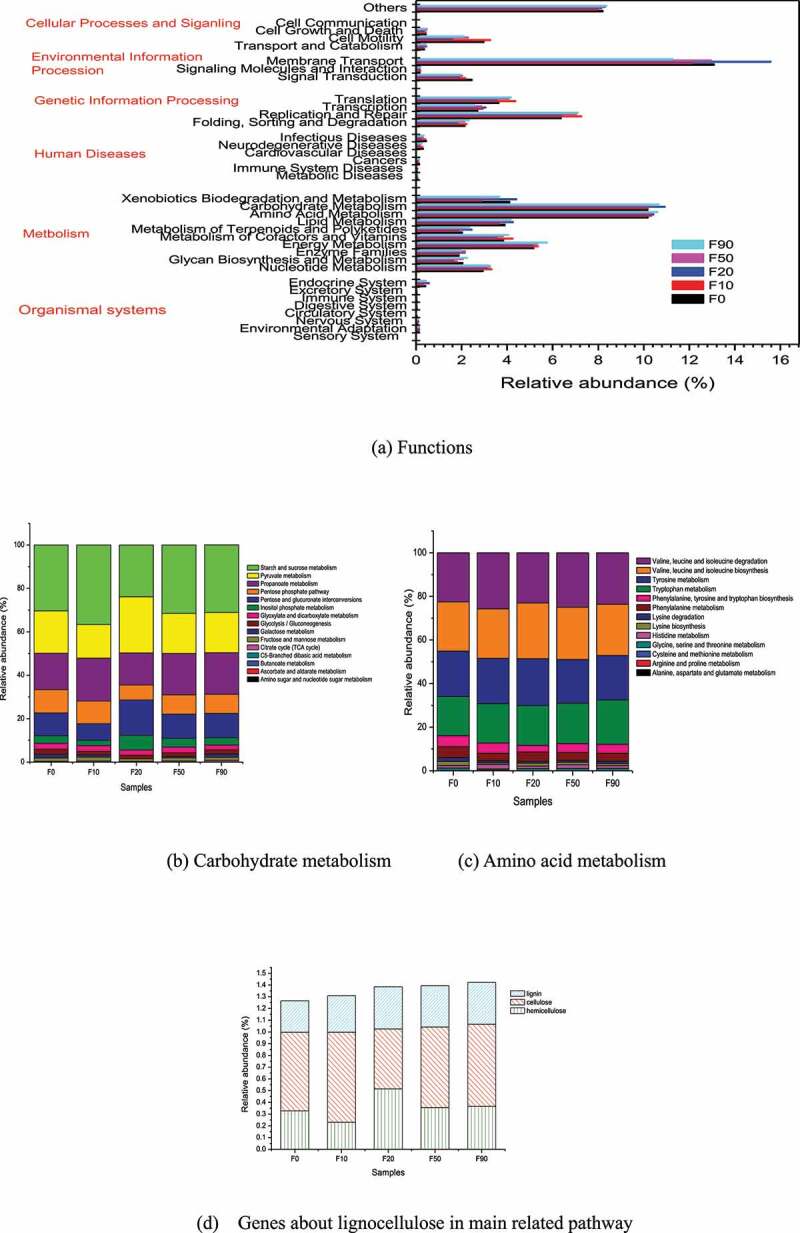
Function predict KO of composting samples

### 3.3. Microbe succession

#### 3.3.1. Phylum

The microbe succession of the stalk compost samples at the phylum level was shown in Figure 4a. 13, 12, 7, 17 and 19 bacterial phyla were detected in F0, F10, F20, F50 and F90, respectively. The number of dominant phyla (relative abundance≥1%) were 4 in F0, 5 in F10, 3 in F20, 6 in F50 and 9 in F90, respectively. The number of phyla and dominant phyla detected in the composting samples were decreased from F0 to F20, then increased from F20 to F90. *Acidobacteria, Chloroflexi, Actinobacteria, Firmicutes, Proteobacteria* and *Bacteroidetes* could be found in these five samples. The relative abundances of *Actinobacteria* and *Firmicutes* in F0, F10, F20, F50 and F90 were high and the total relative abundance was 8.68%, 65.19%, 96.28%, 58.20% and 35.52%, respectively. The relative abundances of *Actinobacteria, Firmicutes, Proteobacteria* and *Bacteroidetes* in F0, F10, F50 and F90 were high, and the total relative abundance of these four bacterial phyla was 99.01%, 98.49%, 91.80% and 85.41% respectively, which decreased obviously. The results were similar to those of Ding et al [14,18]. Except for dominant phyla of *Actinobacteria* (67.14%) and *Firmicutes* (29.13%), *Gemmatimonadetes* (3.43%) were detected as dominant phylum in F20.

The bacterial phyla changed obviously with the increase of composting time. The bacterial phyla with relative abundance above 10% were *Proteobacteria* (64.79%) and *Bacteroidetes* (25.54%) in F0, *Firmicutes* (63.08%) and *Proteobacteria* (19.47%) in F10, *Actinobacteria* (67.14%) and *Firmicutes* (29.13%) in F20, *Actinobacteria* (24.09%), *Firmicutes* (34.11%), *Proteobacteria (*13.31%) and *Bacteroidetes (*20.29%) in F50, and *Actinobacteria* (31.00%), *Proteobacteria* (25.30%) and *Bacteroidetes* (24.59%) in F90. *Actinobacteria* [6], *Firmicutes* [25–28], *Proteobacteria* [27] and *Bacteroidetes* [28] were well-known lignocellulose degrading bacteria, and were abundant during the process of stalk composting process because of high content of lignocellulose in corn stalk [14]. High temperatures had obvious inhibitory effect on *Proteobacteria* and *Bacteroidetes*. And the relative abundances of *Proteobacteria* and *Bacteroidetes* were low in samples F10 and F20 which were at high temperatures above 60°C, and became high in F50 at rather lower temperature than 60°C. *Actinobacteria* and *Firmicutes* can endure high temperature and were abundant in F20 [25,29,30]. The relative abundance of *Actinobacteria* was the most dominant bacteria in F20 at high temperature.

#### 3.3.2. Genus

The microbe succession of the stalk compost samples at the genus level was shown in Figure 4b. 22 genera were totally detected in stalk compost samples. Number of genera were 19, 17, 10, 18 and 18 in F0, F10, F20, F50 and F90, respectively. *Agrobacterium, Brevibacillus, Devosia, Paenibacillus, Coprococcus* and *Pedobacter* were detected in all the five samples. But the dominant genus and relative abundance varied in different sample. Compared with F0, *Coprococcus* (12.20%) and *Ureibacillus* (39.70%) were newly detected, while *Agrobacterium, Enterobacter* and *Janthinobacterium* were not detected yet in F10. The relative abundance of *Sphingomonas, Pseudomonas* and *Pedobacter* decreased from 2.13%, 13.19% and 17.73% in F0 to 1.59%, 4.36% and 1.68% in F10 respectively. The relative abundance of Flavobacterium increased from 1.74% in F0 to 2.34% in F10. Relative abundance of *Ureibacillus* decreased from 39.70% in F10 to 1.09% in F20, *Brevibacillus* became dominant genus with its relative abundance increasing from 0.17% in F10 to 3.07%. *Thermobispora* with relative abundance of 27.19% in F20 was emerged. Compared with F20, *Steroidobacter* (3.09%) and *Paenibacillus* (6.80%) became dominant genera in F50, *Brevibacillus* and *Thermobispora* were still dominant genera, but the relative abundance decreased to 1.90% and 2.90%, respectively. *Paenibacillus, Brevibacillus* and *Thermobispora* were not dominant genera in F90, while *Devosia, Chitinophaga, Streptomyces* and *Nonomuraea* became dominant genera with the relative abundance of 1.89%, 4.51%, 6.61% and 9.56% respectively. *Steroidobacter* still maintained the dominant status with the relative abundance increasing to 10.61%. It can be seen that during the composting process, the dominant genus had undergone significant succession. *Flavobacterium* (1.74%-2.34%), *Pseudomonas* (4.36%-13.19%) and *Pedobacter* (1.68%-17.73%) were dominant genus in F0 and F10. *Ureibacillus* became dominant bacteria in F10 (39.7%) and F20 (1.09%). *Thermobispora* (2.9%-27.12%) was the dominant bacteria in F20 and F50, *Steroidobacter* was the dominant bacteria in F50 and F90.

The bacteria related to the conversion of nitrogen elements such as *Ureibacillus, Sphingomonas, Flavobacterium* and *Pseudomonas* were abundant in sample F10 due to the addition of nitrogen fertilizer. The activities of nitrogen transforming bacteria may lead to the high pH of the system at the beginning stage. *Pseudomonas, Sphingobacteria, Pedobacter, Janthinobacterium* and *Flavobacterium* participated in the degradation of lignocellulose by secreting lignocellulose degrading enzymes [14,30–32]. *Ureibacilluscan* can utilize glucose and xylose produced by cellulose degradation [31]. *Coprococcus* was related to the fermentation of short-chain fatty acids [33]. The relative abundances of *Ureibacillus* and *Coprococcus* were higher than the others due to the existence of protein, starch, amino acid sugars, etc, which were easily hydrolyzed in F10.

The temperature of the compost pile increased and maintained around 70°Cfor the metabolic activities of these bacteria when F20 was sampled [19]. The activities of mesophilic aerobic bacteria such as *Pseudomonas, Flavobacterium, Janthinobacterium, Coprococcus* and *Streptomyces* were inhibited and their relative abundance in F20 were 0 or negligible. Only the thermophilic *Thermobispora, Ureibacillus* and *Brevibacillus* were relatively abundant. *Thermobispora* could secrete endoglucanase (EC:3.2.1.4), beta-glucosidase (EC:3.2.1.21) and 1,4-beta-cellobiosidase (EC:3.2.1.91), which were all related to the biochemical degradation of cellulose [30,34,35]. *Brevibacillus* and *Ureibacillus* could utilize glucose to produce acid [32,36]. The decomposition of cellulose by *Thermobispora* and the utilization of decomposition products by *Brevibacillus* and *Ureibacillus* played key roles at this stage. Therefore pH of the compost pile kept decreasing due to theacid production after the degradation of cellulose.

After the pile was turned over, a large amount of heat was lost. The organic matter in the pile was not enough for bacteria to generate energy to make the pile reach previous high temperature. As a result, the abundance of the thermophilic *Thermobispora* decreased from 27.12% in F20 to 2.90% in F50, while *Paenibacillus, Brevibacillus* and *Steroidobacter* became more abundant than that in F20. *Paenibacillus* was the main bacteria degrading cellulose to glucose by secreting endoglucanase (EC: 3.2.1.4) and beta-glucosidase (EC: 3.2.1.21) [32,37,38], he temperature of composting piles were close to the ambient temperature at the end of composting. Then *Streptomyces, Nonomuraea, Devosia, Steroidobacter* and *Chitinophaga* became dominant in F90. *Streptomyces* participated in the lignocellulose degradation by secreting lignin peroxidase (EC1.11.1.14), manganese peroxidase (EC1.11.1.13), xylanase and cellulase, etc [39–42]. And *Nonomuraea* can degrade hemicellulose [40]. *Chitinophaga* can degrade starch and cellulose, and ferment glucose, fructose and arabinose [33]. It can be seen that the degradation of lignin and hemicellulose also occurred in the maturity stage. This research can support the preparation and application of bacteria agents for corn stalk composting.

Lei et al [43] found the dominant bacteria changed from *Bacillus* and *Ureibacillus* at the early thermophilic stage (3d) to *Bacillus, Thermobifida* and *Norank_f_Limnochordace* during the later thermophilic phase (13d) when the composting materials were swine manure and wheat straws. And the *Norank_o_SBR1031* was main genus both during the cooling phase (23d) and maturation phase (40d). While in this study, *Coprococcus* (12.20%) and *Ureibacillus* (39.70%) were main strains at early thermophilic stage (10d). *Thermobispora* (27.12%) was dominant in thermophilic stage (20d). *Steroidobacter* (3.09%) and *Paenibacillus* (6.80%) were dominant at cooling stage (50d). *Streptomyces* (6.61%), *Nonomuraea* (9.56%) and *Steroidobacter* (10.61%) were dominant during maturation stage (90d). It can be seen that the main microbial community of corn stalk in cold region was different from that of above mentioned paper. It can be explained by the type and volume of substrates leading to different temperatures of composting piles. The temperature of composting pile played an important role on the succession of microbial community during composting. The volume of our composting pile was much larger, which led to the thermophilic, cooling and maturation stage last more days. So there less main microbial community during early thermophilic stage and more main strains during the other composting stages than that in the reference [43].

### 3.4. PICRUSt analysis

#### 3.4.1. KEGG

PICRUSt (phylogenetic investigation of communities by reconstruction of unobserved) is used to predict the metabolic function of bacteria in some process based on the measured 16S rRNA gene sequence and referring to the genome database [24,44]. According to the KEGG Orthology analysis, the bacteria genes in samples F0, F10, F20, F50 and F90 involved 280, 269, 267, 273 and 297 metabolic pathways in level 3, respectively. The gene function of the bacteria can be divided into cellular processes, environmental information processing, genetic information processing, human diseases, metabolism, organic systems, others and unclassified [14,33,42]. The KEGG analysis results of the corn compost were shown in Figure 4a. (2.41 ~ 3.02) % of the genes belonging to the 4 metabolic pathways in cellular processes, (3.39 ~ 17.62) % of the genes were derived from the 3 metabolic pathways in environmental information processing, (14.82 ~ 16.78) % of the genes belonging to genetic information processing among the 4 pathways, (0.62 ~ 1.06) % of the genes were derived from the 6 metabolic pathways in human diseases, (48.06 ~ 51.75) % of the genes were derived from the 10 metabolic pathways in metabolism, (0.69 ~ 0.93) % of the genes might be attributed to the eight metabolic pathways in organic systems, etc. The gene abundance from the same metabolic pathway did not change significantly in the 5 samples, and the abundance related to metabolism was higher. The abundance from different metabolic pathways in the same sample were changed significantly. For example, the abundance of genes related to the organic system varied within 0.69%-0.86%, while those related to metabolism varied from 48.06% to 51.75%. The gene abundance related to environmental information processing were higher than that of genetic information processing in F0 and F20, and the changes were opposite in F10, F50 and F90.

It can be seen from Figure 5a that among the 10 metabolic pathways belonging to metabolis, the metabolic genes related to amino acid metabolism and carbohydrate metabolism were the most, and their abundances were 21.87%~22.71% and 21.69%~22.70%, respectively. Throughout the composting process, the genes from carbohydrate metabolism pathway were related to the degradation of cellulose and hemicellulose [24]. The samples with relative abundance of the genes from carbohydrate metabolism pathway from high to low were F20,  F10,  F50,  F0 and F90. Therefore, the degradation of lignocellulose mainly occurred in the high temperature stage. While, the samples with relative abundance of the genes from amino acid metabolism pathway from high to low were F10,  F50,  F0,  F90 and F20. In the early stage of composting, nitrogen fertilizer was added to adjust the ratio of C/N in the pile, and the protein in the compost material was easily hydrolyzed, which led to the genes from amino acid metabolism pathway which was closely related to the formation of humus increased [44]. Then they declined with the content of protein and fertilizer decreasing as the compost undergoing.

As showed in Figure 5b, the genes of starch and sucrose metabolism, pyruvate metabolism, propanoate metabolism, pentose phosphate pathway, and pentose and glucuronate interconversions from carbohydrate metabolism function during the composting process were abundant. Their relative abundance was account for 23.88%~36.61%, 15.53%~25.83%, 14.78%~19.74%, 6.83%~ 10.73% and 7.68%~16.36% of the total carbohydrate metabolism genes, respectively.

The organisms related to pyruvate metabolism pathway were the most in F20 and the most organisms related to starch and sucrose metabolism pathway werein F10. It was stated that starch and sucrose metabolism mainly occurred during the early stage of composting process. The genes of amino acid metabolism were mainly from valine, leucine and isoleucine degradation, valine, leucine and isoleucine biosynthesis, tyrosine metabolism and tryptophan metabolism with the relative abundances of 22.54%~25.71%, 22.54%~25.61%, 20.01%~21.48% and 17.96%~20.31%, respectively (Seen Figure 5c).

#### 3.4.2. Degradation pathways of lignin and cellulose

During corn stalk composting the degradation pathways of lignin and cellulose could be described according to the map000940 (phenylpropanoid biosynthesis) and map00500 (starch and sucrose metabolism). The degradation pathway of hemicellulose with different structure and molecular units could refer the map000520 (amino sugar and nucleotide sugar metabolism), map000540 (lipopolysaccharide biosynthesi) and map00040 (pentose and glucuronate interconversions). The number of genes in these three hemicellulose pathways was accumulated together. The relative abundance of lignocellulose degrading bacteria was shown in Figure 5d. It can be seen from Figure 5d that the degradation of lignocellulose occurred during the entire composting process. The degradation of cellulose mainly occurred in the early stage at high temperature, and lignin and hemicellulose were mainly degraded in the high temperature stage.

It can be found from the results that the changes in the physicochemical properties of the composting corn stalk were closely related to the metabolic activities of the bacteria. From the beginning of composting to the early stage of high temperature, the metabolism activities of mesophilic bacteria such as *Pseudomonas, Sphingobacteria, Pedobacter, Janthinobacterium* and *Flavobacterium* elevated the temperature of the pile, then these bacteria became inactive or dormant. However the thermophilic bacteria reproduced and became dominant. The composting process was completed by the synergistic action of multiple bacteria. Some bacteria degraded macromolecular organic matter, and some bacteria used the products of degrading bacteria mentioned earlier. Diverse organism cooperated each other to complete the circulation of carbon and nitrogen. *Pseudomonas, Paenibacillus and Streptomyces etc*. degraded lignocellulose into glucose, xylose, and fructose small organic molecules which can be fermented and utilized by *Ureibacillus, Coprococcus, Brevibacillus* and *Chitinophaga*. The pH change of the compost samples was the collective effect of fermentation and ammoniation in different stages. One kind of bacteria can play several roles. *Pseudomonas* not only degraded cellulose, but also participated in denitrification process under aerobic conditions. *Chitinophaga* can not only degrade starch and cellulose, but also use its degradation products [42]. The phylum and genus of lignocellulose degrading bacteria were more diverse and abundant because the corn stalk contains more than 75% lignocellulose. Due to the fact that the composting raw materials and microorganisms all contained nitrogen element, the metabolic pathways were mainly starch, polysaccharides and amino acids.

## 4. Conclusions

The succession and metabolic pathway of lignocellulose degrading bacteria during of corn stalk composting in the cold area were analyzed via 16SrRNA technology. The results showed an obvious microbial succession during corn stalk composting. *Flavobacterium, Pseudomonas* and *Geobacillus* were dominant during early stage. *Ureabacillus* was dominant at early thermophilic stage and thermophilic stage, and *Bisporus* was found to be dominant at high temperature. The bacteria during the composting process of corn stalk were mainly involved in the amino acid metabolism related to the conversion of nitrogen and the Carbohydrate Metabolism related to the degradation of lignocellulose.

## Highlights

- The dominant bacteria changed during the composting process of corn stalk.
- The component of the composting substrate affected the succession of bacteria.
- The bacteria were mainly involved in the amino acid metabolism.
- The lignin and hemicellulose mainly degraded in the high temperature stage.
